# Plasma Exchange for the Treatment of Severe Hypertriglyceridemic Pancreatitis With Diabetic Ketoacidosis: A Case Report From Sub-Saharan Africa

**DOI:** 10.7759/cureus.31311

**Published:** 2022-11-09

**Authors:** Newnex Mongare, Kelvin Orare, Salim Abdallah, Ahmed Sokwala

**Affiliations:** 1 Medicine, Aga Khan University Hospital, Nairobi, KEN

**Keywords:** severe, pancreatitis, hypertriglyceridemia, diabetic ketoacidosis, plasma exchange

## Abstract

The triad of acute pancreatitis, diabetic ketoacidosis, and hyperlipidemia is exceedingly rare. Case reports describing this uncommon triad have successfully been managed with insulin infusions only. Herein, we highlight the challenges in making this diagnosis and present Sub-Saharan Africa’s first experience with therapeutic plasma exchange in the management of hypertriglyceridemic pancreatitis associated with diabetic ketoacidosis, which was initially refractory to insulin infusion alone.

## Introduction

Severe acute pancreatitis (AP) is associated with significant morbidity and mortality, with estimated mortality figures as high as 30-40% in hospitalized patients with attendant organ dysfunction or pancreatic necrosis [1]. Hypertriglyceridemia is the third most common cause of pancreatitis after gallstones and chronic alcohol abuse and is estimated to account for roughly 5% of the cases [2]. Assessment of global temporal trends demonstrates an increasing incidence of hypertriglyceridemic AP (HTGP) because of increasing obesity-related dyslipidemia [3]. Compared to other etiologies of AP, HTGP, particularly with triglyceride (TG) levels above 5.6 mmol/l, has been associated with greater clinical severity and higher complication rates [4]. Furthermore, despite the likeness in the initial management approach to AP regardless of etiology (that is, fluid resuscitation, pain control, and nutritional support), definitive HTGP management may require an insulin or heparin infusion, or therapeutic plasmapheresis [5]. There are no clear evidence-based guidelines on the definitive management of HTGP [6].

There is a paucity of research from Africa on the epidemiology of AP. A South African study [7] demonstrated a similar epidemiological profile to that of the Western world, with dyslipidemias accounting for roughly 8% of the cases. Compared to the West, however, most deaths in the African study tended to occur earlier, emphasizing the need for prompt, and improved supportive and definitive management. With the global epidemiology of dyslipidemia, metabolic syndrome, and diabetes showing a concerning increase especially in the developing world [8], HTGP is bound to become a growing concern in Africa.

To the best of our knowledge, there are no recorded experiences with therapeutic plasma exchange (TPE) for the management of HTGP in Africa. Moreover, there is sparse literature on HTGP presenting together with diabetic ketoacidosis (DKA). Herein, we present our experience with this unique presentation, which was initially unresponsive to insulin infusion, necessitating and responding well to TPE.

## Case presentation

A 38-year-old male not known to have any comorbidities presented to our emergency room with a week's history of polydipsia and polyuria, and a day's history of sudden onset severe epigastric pain that was non-radiating and was associated with nausea and vomiting. The psychosocial history revealed occasional alcohol consumption, which he described as “around 5 beers every weekend” with a score of 0 on the screening CAGE questionnaire. Drug history did not demonstrate a history of non-steroidal anti-inflammatory drug (NSAID) use. There was no known family history of chronic illnesses, including dyslipidemia. On examination, he was alert albeit sick-looking, dehydrated, and visibly tachypneic. His body mass index was 25.5 kg/m2 (Height = 177 cm, Weight = 80 kgs). The blood pressure was 127/66 mmHg, a pulse rate of 110/min, and a respiratory rate of 34/min. There were no physical stigmata of hyperlipidemia. Systemic examination revealed severe epigastric tenderness, with diminished bowel sounds. The chest was clear to auscultation. An initial impression of DKA was made, based on elevated bedside glycemic and ketone levels, with the patient being initiated on an insulin infusion of 0.1 U/kg/h after an initial bolus of 0.1 U/kg along with intravenous fluids.

In the first couple of hours of the patient’s management, there were notable delays in the reporting of some of the patient’s labs with the laboratory requesting multiple repeat sampling due to a “highly lipemic sample.” It was not until approximately twelve hours later that the lab was finally able to process and report the complete first set of labs (marked as day 1) as demonstrated in Table 1.

**Table 1:** Laboratory findings of the case

Investigation / Date	Day 1	Day 2	Day 3	1 month post discharge	Reference range
Total cholesterol	28.12	8.3	6.2	2.06	3-5.5 mmol/L
HDL cholesterol	4.17	0.33	0.2	0.3	> 0.9 mmol/L
LDL cholesterol	10.84	1	1	0.57	< 3.37 mmol/L
Triglycerides	>12.44	>6.22	>6.22	3.21	0.3-1.7 mmol/L
Lipase	1616	1450	1000	56	10-150 U/L
Serum sodium	116	137	141	141	136-146 mmol/L
Serum potassium	4.47	3.59	3	3.52	3.5-4.5 mmol/L
Serum creatinine	111	98	58	49	62-133 µmol/
Serum bicarbonate	<10.0	18	23	26.9	18-22 mmol/L
Blood Urea Nitrogen (Urea)	3.7	3.5	2.3	3.1	1.2-3 mmol/L
WBC count (total)	15.41	8.68	7.8	7.5	4-10 x 10^9/L
Haemoglobin	13.6	8.6	7.4	8.5	13-17 g/d
Platelet count	73	129	172	648	150-400 x 10^9/L
Random blood sugar	27.8	8.1	7.5	6.8	< 6.7 mmol/L
Serum Ketone	3	0.2	0.1	0.1	< 0.6 mmol/L
Serum Ph	7.28	7.34	7.41	7.43	7.35-7.45

**Figure 1:**
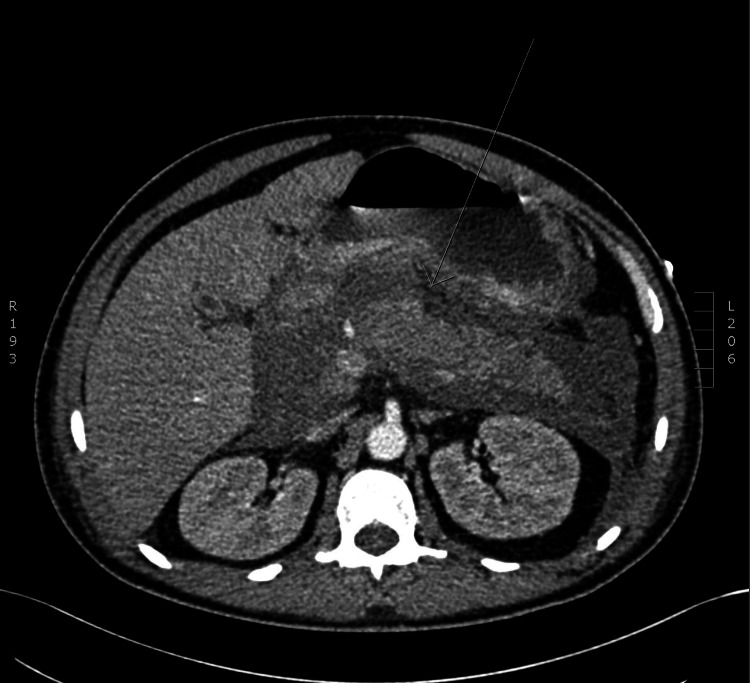
Oedematous pancreas with acute free peripancreatic fluid and no evidence of pseudocyst formation

At this point, the patient had received roughly six liters of intravenous fluid. Glycated hemoglobin (HbA1C) was elevated at 8.3%. A CT abdomen showed an edematous pancreas with acute free peripancreatic fluid and no evidence of pseudocyst formation (Figure 1). 

A diagnosis of severe HTGP was made, with high-dose statin and fenofibrate being added to his management.

**Figure 2:**
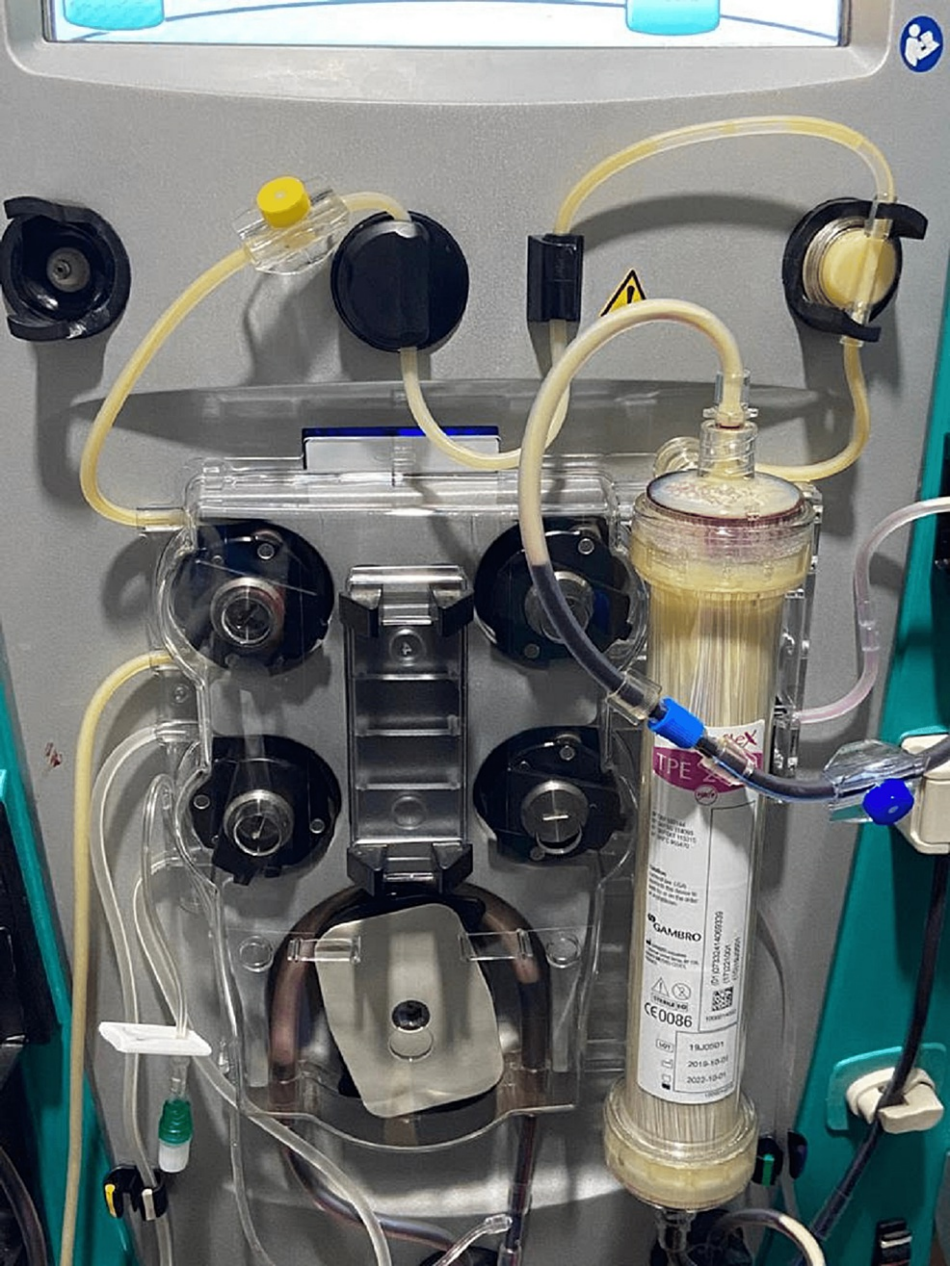
Clogging of plasma exchange by highly lipemic blood

Despite adequate fluid resuscitation and an insulin infusion with an appropriate decline in serum glucose and ketone levels, the patient’s clinical status was now concerning for declining blood pressure and urine output, metabolic acidosis, and pulmonary edema. A decision was made to initiate continuous renal replacement therapy (CRRT), a decision that proved futile due to the highly lipemic blood. To circumvent this, under advisement by the nephrology team, TPE was initiated to periodically interrupt CRRT. The exchange was scheduled to run over 4 hours with a blood flow (Qb) of 200 ml/min to be replaced with 3 liters of fluid constituted as 200 ml of albumin and 800 ml of ringer’s lactate. Heparin was used. The plasma exchange however failed twice due to the clogging of the filter by the highly lipemic blood with a consequent increase in the transmembrane pressures (Figure 2).

TPE was reattempted and completed successfully after its duration was reduced to 2 hours, after which, the patient was able to benefit from CRRT. By the third day of admission, the patient was reporting significant improvement in epigastric pain and better urine output. Serum triglyceride levels had reduced by half after the plasma exchange. There were no additional TPE sessions. The patient went on to be discharged on a basal bolus-insulin regimen, statin and fenofibrate. Two months post-discharge the patient was doing well with adequate glycemic control noted on self-monitoring of blood glucose and an improved lipid profile. He found his inpatient care and outpatient follow-up care satisfactory.

Informed consent was obtained from the patient to use de-identified historical and clinical information.

## Discussion

The triad of AP, DKA, and hyperlipidemia (HL) is rarely seen and only a few cases have been reported so far [9-13]. It has been described as the enigmatic triad with previous authors hypothesizing that DKA is the initial event that leads to transient HL and consequent AP. AP has also been seen in DKA with low triglyceride levels (<3.4 mmol/L), leading these authors to speculate that in the setting of DKA, very low levels of TGs are necessary to cause HTGP or that DKA in itself is a rare cause of AP [10]. Even more enigmatic is the diagnosis of AP in the setting of DKA. For instance, epigastric pain and elevations of serum amylase and lipase levels, which is typical of AP, can occur in isolated DKA as well [14]. Worse, the three-fold increase in serum lipase that is specific for AP diagnosis may be absent in HTGP [15]. Finally, delays in measuring TG levels can lead to falsely low assays, as levels tend to normalize after 72 hours of fasting in HTGP [16]. The management of HTGP patients, as demonstrated in our case, can therefore be complicated by laboratory assay interferences resulting from the high lipemic blood content. This can lead to noteworthy delays in the management of both DKA and HTGP.

Notwithstanding, serum triglyceride levels of more than 11.42 mmol/L are generally considered necessary to induce HTGP, with some population-based studies demonstrating an increased risk of AP by 4% with every 1.142 mmol/L increase in triglycerides [17]. Not every patient with HTG develops AP; HTGP tends to occur in patients with HTG and one or more secondary factors including inadequately controlled diabetes (a possibility in our case), alcoholism, pregnancy, and certain medication (estrogen therapy, olanzapine, protease inhibitors). DKA causes HTG by virtue of being an insulin-deficient state that inhibits lipoprotein lipase (LPL) activity and promotes lipolysis [18]. Lipolysis releases free fatty acids that are ultimately converted to HTG. Certain theories have been proposed to explain the pathophysiology of HTGP [19], the commonest being the effects of elevated levels of free fatty acids on acinar cells, and a capillary plugging process secondary to plasma hyper-viscosity resulting from elevated chylomicron levels. Both these processes lead to ischemia and acidosis, eventually activating trypsinogen and triggering AP.

Definitive HTGP management, therefore, entails activation of LPL activity, such as through insulin infusions, heparin infusions, or fibrates. Plasma exchange has been demonstrated to rapidly reduce serum chylomicrons and triglycerides levels; with some literature quoting about an 80% reduction in TG levels after TPE compared to 40% seen with an insulin infusion. Additionally, TPE has been associated with rapid improvement in clinical symptoms [2], especially epigastric pain as demonstrated in our case as well. There is however no high-quality evidence attributing improved clinical outcomes to TPE, and outcomes have been similar in HTGP patients managed with or without TPE [20].

Case reports describing this triad of AP, DKA, and HL in adults, have been managed entirely by insulin infusion, as theoretically, this modality of treatment potentially addresses both DKA and HTGP. Herein, we present a scenario where clinical deterioration necessitated early utilization of TPE with good response.

## Conclusions

Our case demonstrates the potential challenges in the management of HTGP and highlights the role of TPE in severe HTGP associated with DKA.
